# Prediction of underweight, short stature, and microcephaly based on brain diffusion-weighted imaging sequence in neonates with stage.2 of hypoxic-ischemic encephalopathy: A follow-up study

**DOI:** 10.18502/ijrm.v22i1.15235

**Published:** 2024-02-23

**Authors:** Mohammad Golshan Tafti, Marjan Jafari, Seyed Reza Mirjalili, Razieh Fallah, Farimah Shamsi

**Affiliations:** ^1^Department of Pediatrics, Ali-ebn-Abitaleb School of Medicine, Islamic Azad University, Yazd Branch, Yazd, Iran.; ^2^Department of Neonatology, Shahid Beheshti Hospital, Isfahan University of Medical Sciences, Isfahan, Iran.; ^3^Department of Neonatology, Mother and Newborn Health Research Center, Shahid Sadoughi University of Medical Sciences, Yazd, Iran.; ^4^Department of Pediatrics, Children Growth Disorder Research Center, Shahid Sadoughi University of Medical Sciences, Yazd, Iran.; ^5^Center for Healthcare Data Modeling, Department of Biostatistics and Epidemiology, School of Public Health, Shahid Sadoughi University of Medical Sciences, Yazd, Iran.

**Keywords:** Hypoxic ischemia encephalopathy, Magnetic resonance imaging, Diffusion weighted imaging, Microcephaly, Underweight.

## Abstract

**Background:**

Hypoxic-ischemic encephalopathy (HIE), caused due to reduced oxygenation and brain blood flow, occurs in 1-8 per 1000 live full-term births in developed countries and up to 26 per 1000 live in the developing world. The growth status of survivors of birth HIE has not been evaluated sufficiently.

**Objective:**

This study evaluated, the growth parameters (weight, height, and head circumference) of neonates with Sarnat stage.2 of HIE at 6, 10, and 12 months and its relationship with findings of neonatal brain diffusion-weighted imaging (DWI) sequence.

**Materials and Methods:**

Medical records and growth parameters of 35 neonates with gestational age > 34 wk who were admitted with stage.2 of HIE in Neonatal Intensive Care Unit of Shahid Sadoughi hospital, Yazd, Iran from March 2021-March 2022, and its relationship with neonatal brain DWI sequence finding was evaluated.

**Results:**

15 girls and 20 boys with a mean birth weight of 2880.3 ± 221.8 gr were evaluated. Conventional magnetic resonance imaging and DWI were found to be abnormal in 6 (17.1%) and 18 neonates (51.4%). The most abnormal finding of DWI was high signal in basal ganglia/thalamus in 9 neonates (25.7%). Abnormal DWI is more frequent in neonates with seizures and low birth weight. Hospital stay days were more prolonged in neonates with abnormal DWI. Microcephaly at 12 months was more frequent in children with abnormal DWI.

**Conclusion:**

In survivors of moderate neonatal HIE, abnormal brain DWI sequence might predict inappropriate head growth, and need close medical and nutritional interventions for growth improvement.

## 1. Introduction

Reduced oxygenation and brain blood flow defines as hypoxic-ischemic encephalopathy (HIE), occurs in 1–8 per 1000 live full-term births in developed countries and up to 26 per 1000 live full-term births in the developing world. Depending on HIE stage, 20–30% die in neonatal, 15–20% die in the postnatal period, and severe and permanent neuropsychological sequelae and neurodevelopmental delay may occur in 25–50% of survivors. After birth, severity of neonatal encephalopathy is determined based on clinical signs by Sarnat staging of HIE (Table I) (1).

**Table 1:** Sarant staging of HIE


**Signs**	**Mild HIE (stage.1)**	**Moderate HIE (stage.2)**	**Severe HIE (stage.3)**
**Level of consciousness**	Hyperalert	Lethargic	Stuporous, coma
**Muscle tone**	Normal	Hypotonic	Flaccid
**Posture**	Normal	Flexion	Decerebrate
**Tendon reflexes/clonus**	Hyperactive	Hyperactive	Absent
**Myoclonus**	Present	Present	Absent
**Moro reflex**	Strong	Weak	Absent
**Pupils**	Mydriasis	Miosis	Unequal, poor light reflex
**Seizures**	None	Common	Decerebration
**Electroencephalographic findings**	Normal	Low voltage changing to seizure activity	Burst suppression to isoelectric
**Duration**	< 24 hr if progresses; otherwise, may remain normal	24 hr to 14 days	Days to weeks
**Outcome**	Good	Variable	Death, severe deficits
HIE: Hypoxic-ischemic encephalopathy			

Today, magnetic resonance imaging (MRI) and diffusion-weighted imaging (DWI) sequence are the most sensitive and non-invasive imaging modalities for detecting hypoxic brain injury in newborns. Although, such injury can be detected at various times and with varying pulse sequences, diffusion-weighted sequences during the 1 ${\;}^{\text{st}}$ wk of life are necessary to identify acute injury (2–4). Since symptoms can develop over days, serial neurologic examinations and evaluation of tonicity are essential (4).

Therapeutic hypothermia, within the first 6 hr after birth and if maintained for 72 hr, decreases mortality and major neurodevelopmental impairment at 18 months of age (5).

The prognosis of HIE depends on the timing and severity of the incident and the treatment. The outcome in stage.1 of Sarnat staging of HIE is good and stage.3 of HIE with severe encephalopathy (flaccid coma, apnea, absence of oculocephalic reflexes, refractory seizures) is considered as poor prognosis; however, the neurological outcome in stage.2 of HIE varies (1, 6). Therefore, in all neonates with moderate to severe HIE, developmental assessment for early detection of neurodevelopmental delay and prompt referral for developmental, rehabilitative, and neurologic early intervention services is necessary to achieve the best possible outcomes (1, 7).

In many research studies, survivors of moderate to severe HIE neurodevelopment outcomes were evaluated. However, growth parameters are good indicators of children's general health, and there are currently fewer studies on the growth prognosis of survivors of moderate HIE (8–10).

In the present study, growth parameters (weight, height, and head circumference) of neonates with stage.2 of HIE at 6, 10, and 12 months of age and its relationship with findings of DWI sequence of neonatal brain MRI were evaluated.

## 2. Materials and Methods

In this follow-up study, medical records, non-contrast brain MRI and its DWI sequence of neonates with stage.2 of HIE who were admitted to the neonatal intensive care unit of Shahid Sadoughi hospital, Yazd, Iran from March 2021 to March 2022 were evaluated. Multiple pregnancies, major congenital anomalies and chromosomal abnormalities, severe systemic disease, other intracranial pathology, known contraindications to MRI and presence of any metal prosthesis in their body, manifestations of central nervous infections and Covid-19 infection, and neonates with traumatic delivery were excluded.

Care was taken to include all neonates with ≥ 34 wk of gestational age who were admitted in neonatal intensive care unit with stage.2 of HIE and those whose MRI was done during the 1 ${\;}^{\text{st}}$ wk of life in Shahid Sadoughi hospital, Yazd, Iran. None of the study participants received therapeutic hypothermia.

All neonates underwent standard protocol and conventional MRI (Magetom-Avanto 1.5T magnetic resonance scanner, Simens, Erlangen, Germany). The standard protocol includes T1 weighted sagittal, T2 weighted axial, fluid attenuated inversion recovery axial, and DWI apparent diffusion coefficient. A radiologist interpreted non-contrast brain MRI of these children. All neonates and their growth parameters (weight, height, and head circumference) at the ages of 6, 10, and 12 months were evaluated by the pediatric resident of research.

“All babies were weighted by a digital weighing scale with a sensitivity of 10 gr without diapers. The weighting scale was calibrated at regular intervals. The supine crown heel length was measured on the infantometer with the help of an assistant to the nearest millimeter in the recumbent position. Head circumference was measured using a flexible, non-stretchable tape measure, which runs from the supraorbital ridge to the occiput in the path as the maximum occipitofrontal circumference. To obviate errors due to inter-observer variations, all measurements were made in the Pediatric Neurology Clinic of Shahid Sadoughi hospital by the pediatric resident of research. For the assessment of growth parameters (height and weight), the World Health Organization growth charts were used” (1).

Weight below the 3 ${\;}^{\text{rd}}$ percentile on a standard growth curve was considered underweight, as reached 40 postmenstrual weeks. A height of < 2 standard deviations “from height age was considered short stature (1), and head circumference of > 2 standard deviations below the mean for a given age, sex, and gestation was considered microcephaly based on definition of the American academy of neurology practice parameter (11). The same children were evaluated at 6, followed up to 12 months, and their growth parameters were evaluated at 10 and 12 months”.

### Sample size

Sampling will be available until the completion of the sample size, which is based on the following formula: 


$$n=\frac{Z_{1-\frac{\alpha }{2}}P(1-P)}{d^{2}}$$

Considering the confidence level of 95% and the power of the test is 80% and the type one error (alpha) is equal to 5% and P (the proportion of infants with hypoxic ischemic encephalopathy of the second stage who have developmental delay is equal to 50% based on previous studies) and the value of d or the usual error ratio is equal to 10 about 50% was determined. non-random, easy to complete the sample size and data collection technique of examining the hospitalization records of newborns and completing the relevant questionnaire based on the findings recorded in the newborn's file and information about the research variables including the baby's age, sex, birth weight, gestational age of the baby. The results of physical examination, paraclinical tests and MRI (conventional) and brain (DWI sequence) results. Finally, the information about the research variables entered in the relevant questionnaire, after completing the questionnaire, the statistical analysis of the data was done.

### Ethical considerations

The Ethics Committee of Shahid Sadoughi University of Medical Sciences, Yazd, Iran approved this study (Code: IR.SSU.MEDICINE.REC.1399.272). Before enrolling in the study, written informed consent was obtained from the parents of the infants.

### Statistical analysis

The data were presented by mean and standard deviation for continuous variables, and frequency and percent for qualitative variables. The Chi-square test was used for data analysis of qualitative variables, and mean values were compared using student *t* test. Generalized equation estimation (GEE) method was used to evaluate the changes in weight, height, and head circumstances during the follow-up time based on DWI categories. The Statistical Package for the Social Sciences (IBM SPSS Statistic Version 21, IBM Inc., Chicago, IL) was used to analyze the data and all statistical tests were considered significant at the level of 0.05.

## 3. Results

In this study, 38 babies (including 15 girls and 20 boys) were examined. Parents of 3 infants did not return for follow-up; therefore, their infants were excluded from the study. Finally, 35 infants, including 15 girls (42.8%) and 20 boys (57.2%), with a mean gestational age of 37.1 ± 1.3 wk and mean birth weight of 2880.3 ± 221.8 gr, were evaluated.

13 infants (37%) were premature (gestational age ≤ 37 wk), and 2 infants (6%) were post term (gestational age ≥ 42 wk).

The delivery was cesarean section in 21 neonates (60%), and 8 neonates had birth weight ≤ 2500 gr (low birth weight).

Conventional MRI was abnormal in 6 neonates (17.1%), and DWI sequence was abnormal in 18 neonates (51.4%), and abnormal results in DWI included:

- High signal in T1W and fluid-attenuated inversion recovery in basal ganglia/thalamus in 9 neonates

- Cerebral cortical high signal in T1W in the temporal lobe in 4 neonates

- Decreased diffusion (increased signal) in parasagittal cerebral cortex and subcortical white matter in 3 neonates

- Increased signal in T2W of periventricular white matter in 2 neonates

The result of the DWI sequence of brain MRI based on neonates' characteristics is illustrated in table II, which indicated that abnormal DWI was more frequent in neonates with seizures and birth weight < 2500 gr. No statistically significant differences were observed from viewpoints of sex distribution, route of delivery, gestational age, and birth microcephaly in both groups.

A comparison of means of gestational age, birth weight, birth head circumference, and hospital stay days based on DWI sequence result are shown in table III, indicating that hospital admission duration was more prolonged in neonates with abnormal DWI. However, other means were not statistically significantly different between 2 groups.

Different growth parameter disorders were evaluated at 6, 10, and 12 months. Results showed that obesity and macrocephaly were not seen in any infant. Table IV showed the frequency of underweight, short stature, and microcephaly at 6, 10, and 12 months of age, which illustrated that the frequency of these growth disorders were not statistically significant. In addition, a comparison of the frequency of underweight, short stature, and microcephaly at months 6, 10, and 12 based on the result of the DWI sequence of brain MRI are shown in table IV, which indicated microcephaly at 12 months was more frequent in children with abnormal neonatal DWI sequence, but underweight and short stature were not found to be statistically significant in 2 groups (normal or abnormal DWI).

GEE method with linear distribution was used to assess the significant changes in weight, height, and the head circumstances of neonates during follow-up time based on the result of DWI sequence of brain MRI. The presented results of the longitudinal analysis in table V illustrated that no significant interaction was observed between DWI abnormality and the time of follow-up. However, considering the height of neonates, the abnormality in DWI was marginally significant during follow-up time that was showed in table IV.

**Table 2:** Frequency distribution of diffusion weighted imaging, microcephaly sequence result based on neonates' characteristics


**Variables**		**Normal (n = 17)**	**Abnormal (n = 18)**	**P-value**
Sex*				
	**Girl**	8 (47.05)	7 (38.8)	
	**Boy**	9 (52.95)	11 (61.2)	0.60
Route of delivery*				
	**Vaginal**	7 (41.1)	7 (38.8)	
	**Cesarean**	10 (58.9)	11 (61.2)	0.90
Neonatal seizure*		5 (14)	15 (83.3)		0.001
Low birth weight*		1 (5.8)	7 (38.8)	0.01
Birth microcephaly*		1 (5.8)	0 (0)	0.50
Gestational age (wk)**		36.5 ± 2.06	36.7 ± 1.78	0.50
Birth weight (gr)**		2691.2 ± 661.1	3072.2 ± 461.7	0.10
Birth head circumference (cm)**		33.94 ± 0.9	34.25 ± 0.86	0.70
*Data presented as n (%). Chi-square test. **Data presented as Mean ± SD. *t* test				

**Table 3:** Comparison of prematurity and hospital stay days based on diffusion weighted imaging, microcephaly sequence result


**Variables**	**Normal (n = 17)**	**Abnormal (n = 18)**	**P-value**
Prematurity*	8 (47)	5 (27.7)	0.50
Hospital stay in days**	28.65 ± 11.31	44.44 ± 13.4	0.01
*Data presented as n (%). Chi-square test. **Data presented as Mean ± SD. *t *test			

**Table 4:** Comparison of frequency of underweight, short stature, and microcephaly at months 6, 10, and 12 based on result of diffusion weighted imaging, microcephaly sequence of brain MRI


**Growth disorder**		**Total**	**Normal (n = 17)**	**Abnormal (n = 18)**	**P-value † **
**Underweight**					
	**6 months**	7 (2)	2 (11.7)	5 (27.7)	0.20
	**10 months**	8 (22)	3 (17.6)	5 (27.7)	0.50
	**12 months**	3 (8.6)	1 (5.8)	2 (11.1)	0.60
	P-value = 0.2**				
**Short stature**					
	**6 months**	9 (25.7)	2 (11.7)	7 (38.8)	0.06
	**10 months**	9 (25.7)	2 (11.7)	7 (38.8)	0.06
	**12 months**	5 (14.3)	1 (5.8)	4 (22.2)	0.20
	P-value = 0.4**				
**Microcephaly**					
	**6 months**	13 (37)	5 (29.4)	8 (44.4)	0.30
	**10 months**	14 (40)	5 (29.4)	9 (50)	0.20
	**12 months**	11 (31.4)	3 (17.6)	8 (44.4)	0.01
	P-value = 0.7**				
Data presented as n (%). **Chi-square test. † Fisher's exact-test. MRI: Magnetic resonance imaging					

**Table 5:** Estimated coefficients and standard errors considering weight, height, and head circumstances at months 6, 10, and 12 based on result of DWI sequence of brain MRI


**Variables**			**B**	**St. Error**	**95% CI**	**P-value**
**Weight**						
	**DWI**					
	**Normal**	- -	- -
	**Abnormal**	0.26	0.2	-0.12, 0.65	0.184
	**Time**					
	**6 months**	- -	- -
	**10 months**	1.46	0.10	1.25, 1.67	< 0.001
	**12 months**	2.58	0.18	2.21, 2.95	< 0.001
	**DWI × time**					
	**Abnormal × 10 months**	0.04	0.17	-0.3, 0.37	0.821
	**Abnormal × 12 months**	0.23	0.29	0.34, 0.8	0.434
**Height**						
	**DWI**					
	**Normal**	- -	- -
	**Abnormal**	1.10	0.59	2.27, 3.42	0.064
	**Time**					
	**6 months**	- -	- -
	**10 months**	4.67	0.37	3.94, 5.39	< 0.001
	**12 months**	7.11	0.42	3.94, 5.39	< 0.001
	**DWI × time**					
	**Abnormal × 10 months**	0.07	0.53	-0.90, 1.04	0.89
	**Abnormal × 12 months**	0.09	0.49	-0.95, 1.13	0.86
**Head circumstances**						
	**DWI**					
	**Normal**	- -	- -
	**Abnormal**	0.35	0.59	-0.82, 1.52	0.56
	**Time**					
	**6 months**	- -	- -
	**10 months**	1.69	0.12	2.48, 3.02	< 0.001
	**12 months**	2.57	0.13	1.45, 1.94	< 0.001
	**DWI × time**					
	**Abnormal × 10 months**	0.16	0.18	-0.19, 0.51	0.38
	**Abnormal × 12 months**	0.19	0.21	-0.23, 0.61	0.37
GEE method with linear distribution used. DWI: Diffusion-weighted imaging, MRI: Magnetic resonance imaging						

## 4. Discussion

In the present study, growth parameters (weight, height, and head circumference) of neonates with stage.2 of hypoxic ischemic encephalopathy at 6, 10, and 12 months and its relationship with findings of DWI sequence of neonatal brain MRI were evaluated.

Risk of neurodevelopmental delay and moderate to severe cerebral palsy is higher in infants with moderate or severe hypoxic ischemic encephalopathy (12). In children with moderate to severe cerebral palsy, many factors such as discoordination of oropharyngeal muscles and suboptimal nutritional intake, recurrent aspiration, chronic reflux, increased caloric consumption because of extreme muscle contraction in spasticity and growth hormone deficiency may be responsible for sluggish growth velocity in these children (13, 14).

In the present study, the frequency of underweight and short stature at 6, 10, and 12 months was not different between groups (children with normal or abnormal neonatal brain DWI sequence). But, in a study, the frequency of slow growth in children with moderate to severe cerebral palsy was more than those with no cerebral palsy at 18–22 months and 6–7 yr and increasing severity of slow growth was associated with increasing age (13). In a study in India, anthropometric parameters of neonates with gestational age ≥ 36 wk with moderate to severe HIE were evaluated. “All anthropometric parameters were significantly lower in the presence of death or disability at 1 yr of age and weight gain were associated with better neurodevelopmental outcomes at 1 yr of age”, and they concluded that nutritional interventions may potentially amend the prognosis of neonates with HIE (15, 16).

In the present study, microcephaly at 12 months was more frequent in infants with abnormal neonatal DWI sequence. But, in a study in London, the head growth of neonates with HIE in the first year of life were compared with the control group. At 12 months, 48% of infants with HIE and 3% of the control group had microcephaly, while suboptimal head growth was seen in 53% of HIE infants and 3% of the controls. Suboptimal head growth was more frequent in neonates with severe white matter and basal ganglia and thalamus involvement in their brain MRI (17).

In the present study, abnormal DWI is more frequent in neonates with seizures. In a study the outcome of 208 neonates with HIE and their relationship with clinical seizures during the first 4 days of life was evaluated at 18 months. Regardless of the severity of HIE and treatment protocol, seizures were not associated with death or moderate to severe disability. The authors concluded that mortality and morbidity in neonates with HIE and seizure can be better explained by the underlying severity of encephalopathy (18).

Based on the present study, association, and relationship between the result of neonatal brain DWI sequence and growth disorders such as underweight, obesity, short stature, and macrocephaly were not found. However, microcephaly and poor head circumference growth at 12 months was more frequent in children with abnormal neonatal DWI sequence.

## 5. Conclusion

In survivors of moderate neonatal HIE, abnormal brain DWI sequence and white matter and basal ganglia involvement might predict inappropriate and improper head growth. Close medical and nutritional interventions may be useful in the improvement of their growth.

##  Data availability

Data supporting the findings of this study are available upon reasonable request from the corresponding author.

##  Author contributions

Razieh Fallah and Marjan Jafari designed the study and conducted the research. Seyed Reza Mirjalili and Farimah Shamsi monitored, evaluated, and analyzed the result of the study. Further, Mohammad Golshan reviewed the article. All authors approved the final manuscript and take responsibility for the integrity of the data.

##  Conflict of Interest

The authors declare that there is no conflict of interest.
